# Direct and Sensitive Electrochemical Determination of Total Antioxidant Capacity in Foods Using Nanochannel-Based Enrichment of Redox Probes

**DOI:** 10.3390/molecules29112423

**Published:** 2024-05-21

**Authors:** Lixia Duan, Chaoyan Zhang, Fengna Xi, Danke Su, Wenhao Zhang

**Affiliations:** 1 Guangxi Medical University Cancer Hospital, Guangxi Medical University, Nanning 530021, China; duanlixia@sr.gxmu.edu.cn (L.D.); sudanke33@sina.com (D.S.); 2 School of Chemistry and Chemical Engineering, Zhejiang Sci-Tech University, Hangzhou 310018, China; 202130107346@mails.zstu.edu.cn

**Keywords:** total antioxidant capacity, electrochemical determination, silica nanochannel film, colored sample, food sample

## Abstract

Simple and sensitive determination of total antioxidant capacity (TAC) in food samples is highly desirable. In this work, an electrochemical platform was established based on a silica nanochannel film (SNF)-modified electrode, facilitating fast and highly sensitive analysis of TAC in colored food samples. SNF was grown on low-cost and readily available tin indium oxide (ITO) electrode. Fe^3+^-phenanthroline complex-Fe(III)(phen)_3_ was applied as the probe, and underwent chemical reduction to form Fe^2+^-phenanthroline complex-Fe(II)(phen)_3_ in the presence of antioxidants. Utilizing an oxidative voltage of +1 V, chronoamperometry was employed to measure the current generated by the electrochemical oxidation of Fe(II)(phen)_3_, allowing for the assessment of antioxidants. As the negatively charged SNF displayed remarkable enrichment towards positively charged Fe(II)(phen)_3_, the sensitivity of detection can be significantly improved. When Trolox was employed as the standard antioxidant, the electrochemical sensor demonstrated a linear detection range from 0.01 μM to 1 μM and from 1 μM to 1000 μM, with a limit of detection (LOD) of 3.9 nM. The detection performance is better that that of the conventional colorimetric method with a linear de range from 1 μM to 40 μM. Owing to the anti-interfering ability of nanochannels, direct determination of TAC in colored samples including coffee, tea, and edible oils was realized.

## 1. Introduction

Oxidative stress is closely related to health. Oxidative stress refers to the accumulation of excessive free radicals or oxidizing substances in the body, exceeding the body’s natural defense mechanisms, potentially causing harm to human health [1]. For instance, oxidative stress is associated with many diseases, especially cardiovascular diseases, cancer, and diabetes. Additionally, oxidative stress can lead to oxidative damage in cells and tissues and contribute to the aging process [2]. To combat oxidative stress, the human body relies on antioxidant systems, which include endogenous antioxidants and antioxidants obtained through dietary intake [3,4]. Commonly, natural foods are rich in antioxidants, such as vitamin C (VC), vitamin E, carotenoids, and polyphenols. In addition, antioxidants also serve as essential food additives. Thus, consuming foods abundant in antioxidants can help maintain the balance between oxidation and antioxidation, reducing the risk of diseases [4]. The development of selective and sensitive techniques for measuring the antioxidant capacity of foods has become increasingly important.

The total antioxidant capacity (TAC) reflects the comprehensive antioxidant ability of all antioxidants in a sample, and it is a crucial indicator for measuring the overall antioxidant content in food [5,6,7]. Specifically, a higher TAC value indicates that the sample has a stronger ability to resist oxidative stress and free radical damage, contributing to maintaining health and reducing the risk of diseases. Achieving accurate and rapid determination of TAC in food is of great significance. Traditional instrumental analysis methods such as high-performance liquid chromatography (HPLC), gas chromatography (GC), and surface-enhanced Raman spectrum (SERS) can accurately measure the types and concentrations of antioxidants [8,9,10,11]. However, these methods are often limited by their expensive equipment, time-consuming operations, and the need for specialized technical expertise. Colorimetric methods for TAC determination have also been developed [12]. For instance, analysis based on phenanthroline (phen), 2,2′-azino-bis(3-ethylbenzothiazoline-6-sulfonic acid (ABTS) radical cation, or 2,2-diphenyl-1-picrylhydrazyl (DPPH) has been developed [13,14]. Although colorimetric analysis is easy to operate, it cannot directly detect turbid or colored samples. Recently, electrochemical detection has gained widespread attentions. Electrochemical sensors offer advantages such as low equipment cost, high determination sensitivity, rapid response, and ease of miniaturization and integration [15,16,17]. Electrochemical sensors have been utilized for the assessment of TAC in different samples [18,19]. For instance, TAC in herb or beverage has been quantified utilizing a nanocomposite-graphene/poly (3,4-ethylenedioxythiophene): poly (styrenesulfonate)-modified screen-printed carbon electrode (G/PEDOT:PSS/SPCE) based on DPPH assay [18]. Multi-walled carbon nanotubes (MWCNTs)-modified electrode was also employed for the determination of TAC in berries using amperometric tyrosinase biosensor [19]. However, when detecting samples with complex matrices, electrodes are susceptible to contamination. On the one hand, biomolecules in the sample (such as proteins, DNA, RNA) or large particles tend to non-specifically adsorb to the electrode surface, thereby reducing the determination sensitivity and accuracy [20,21,22]. On the other hand, the presence of redox species in the sample can lead to interference in the electrochemical signals [23]. Therefore, the development of electrochemical sensor for direct analysis of TAC in food samples without complex sample pretreatment crucial.

Using a barrier film on the electrode surface is an effective means to reduce or eliminate the influence of sample matrices, thus maintaining the stability and accuracy of electrode [24,25,26]. In recent years, porous materials have gained significant attention due to their high surface area and size-selective capabilities [27,28,29,30]. Silica nanochannel film (SNF) is a nanoscale ultrathin film with a thickness that can be adjusted between 20 to 200 nm [31,32]. It has a vertically aligned and ordered array of nanochannels with uniform nanochannel diameter (typically ranging from 2 to 3 nm) and a high pore density (approximately 75,000 nanochannels/cm^2^) [33]. As a silica thin film, SNF possesses good mechanical stability and is highly compatible with 2D planar electrode, making it suitable for modification of the electrode [34,35]. Additionally, the ultrasmall nanochannel of SNF offers selective sieving capability at the molecular level for the supporting electrode [36,37,38]. On the one hand, SNF-modified electrodes exhibit outstanding resistance to contamination, making them promising for direct electrochemical analysis of complex samples [39,40,41]. This is attributed to the electrical insulating properties of silica material and the size-selective function of its ultrasmall nanochannels, which only permit small molecules to pass through, effectively excluding larger biological molecules and solid particles [42,43,44]. In addition, SNF-modified electrodes possess charge-selective permeability. The nanoscale thickness and ultrahigh pore density of SNF ensure efficient diffusion and mass transport. However, unlike microscale pores, nanochannels also exhibit charge-selective capabilities at the molecular level, allowing only small molecules with matching charges to pass through [23]. This significantly reduces interference from co-existing electroactive substances and enables the enrichment of specific probe or analytes [45,46,47]. For instance, SNF contains numerous silanol groups with a p*K*_a_ value of approximately 2, and the negatively charged surface generated after ionization can electrostatically interact with positively charged analytes, leading to remarkable enrichment [48,49,50]. Therefore, SNF can preconcentrate small analytes or electrochemical probes with opposite charges and reduce interference or fouling from matrix in complex samples.

In this work, a sensitive electrochemical sensor for the determination of total antioxidant capacity (TAC) in foods was demonstrated based on SNF-modified electrode. SNF was grown on a low-cost and readily available indium-tin oxide (ITO) electrode. When 6-hydroxy-2,5,7,8-tetramethylchroman-2-carboxylic acid (Trolox) was employed as the model antioxidant, the Fe^3+^-phenanthroline complex (Fe(III)(phen)_3_) probe can react with Trolox to produce Fe^2+^-phenanthroline complex (Fe(II)(phen)_3_), leading to electrochemical signal at a constant oxidation potential. Based on this principle, the determination of the concentration of antioxidants can be achieved by measuring the oxidation current corresponding to the produced Fe(II)(phen)_3_. The enrichment of Fe(II)(phen)_3_ based on charge-based permeability of nanochannels and the resulting enhancement in detection sensitivity were investigated. The anti-interference capability of the SNF/ITO electrode owing to size-exclusion effect of nanochannels was studied. The detection performance of the fabricated sensor for TAC determination in colored food samples such as coffee, tea, and sesame oil was assessed.

## 2. Results and Discussion

### 2.1. Construction of An Nanochannel-Modified Electrode for Electrochemical Determination of TAC

**Figure 1:**
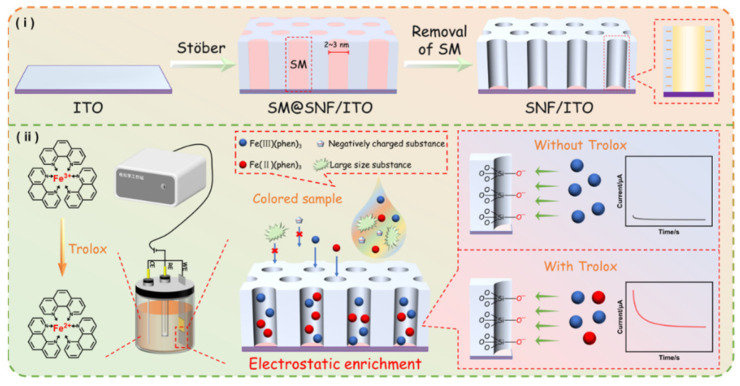
Schematic illustration for the preparation of the SNF-modified ITO electrode (**i**) and electrochemical determination of TAC (**ii**) using nanochannel-based enrichment of Fe(II)(phen)_3_.

In this work, an electrochemical platform was established for detection the total antioxidant capacity (TAC) of food samples using a SNF-modified electrode with Fe(III)(phen)_3_ as the probe. As illustrated in Figure 1, a layer of SNF was grown on the surface of the ITO electrode using the Stöber solution growth method, which can realize the preparation of large-area SNF film with long-range order and excellent coverage [51]. Typically, the positively charged cationic surfactant, hexadecyltrimethylammonium bromide (CTAB), is adsorbed onto the negatively charged ITO and assembles into spherical micelles (SM). Subsequently, under the influence of ammonia and ethanol, siloxane precursor (TEOS) undergoes hydrolysis, forming negatively charged silicate oligomers, that further condense around the SM template. As ethanol molecules diffuse into the CTAB micelles, the alkyl interactions at the tail end of the micelles weaken, leading to an increase in micelle volume and the formation of cylindrical micelles. Then, the resulting SNF has vertically aligned nanochannels sealed by SM on the supporting ITO electrode (SM@SNF/ITO). A washing step in hydrochloric acid–ethanol solution is employed to remove the SM, resulting in electrode with open nanochannels (SNF/ITO). Due to the abundant presence of silanol groups (Si-OH) with low p*K*_a_ (~2), SNF carries a net negative charge in commonly used medium. Coupled with high surface area of SNF, SNF/ITO can significantly enrich positively charged redox probes.

Currently, TAC can ben detected based on the colorimetric assay using phenanthroline, where the mechanism involves the reduction of Fe^3+^ to Fe^2+^ by antioxidants in the sample. The resulting Fe^2+^ forms a stable orange-red complex with phenanthroline (Fe(II)(phen)_3_), generating a characteristic absorption peak at 520 nm. By measuring the absorbance of Fe(II)(phen)_3_ at 520 nm, the TAC in the sample can be calculated. Inspired by this method, the electrochemical oxidation of Fe(II)(phen)_3_ is measured to correlate with the antioxidant content in samples, thus establishing an electrochemical method for TAC detection. Specifically, to achieve electrochemical determination of TAC, Fe(III)(phen)_3_ was used as the probe, which can be chemically reduced to Fe(II)(phen)_3_ by antioxidants. When the model antioxidant Trolox was present, the conversion from Fe(III)(phen)_3_ to Fe(II)(phen)_3_ was controlled by the redox potential of Fe^3+^/Fe^2+^ in presence of phenanthroline ligand. Since Fe^2+^ (with a d^6^ electron configuration) is a proficient cationic electron donor, phenanthroline ligand with low-lying vacant antibonding orbitals can form strong complexes with it, whereas Fe^3+^, due to its higher charge, has inferior electron-donating properties. Consequently, phenanthroline forms a stronger and more stable complex with Fe^2+^. Clearly, the content of Trolox is correlated with the generation of Fe(II)(phen)_3_. In other words, the more antioxidants present, the greater the amount of Fe(II)(phen)_3_ produced. To distinguish the two substances, chronoamperometry (current–time curve, *I*–t) was employed when different potentials were applied. Specifically, when the potential is set to the oxidation potential, the Fe(II)(phen)_3_ is oxidized, resulting in a current signal, allowing for the quantitative determination of Fe(II)(phen)_3_, and consequently the quantification of Trolox. In addition, the SNF/ITO electrode, which significantly enriches Fe(II)(phen)_3_, enhances the current as well as the detection sensitivity. Due to the size-exclusion effect of the ultrasmall nanochannels in SNF, large macromolecules or particles can not interfere the determination. Combined with the advantage of electrochemical determination that is not affected by sample color, the constructed sensor can determination TAC in color or tumid samples. It also eliminates the need for long incubation and sample pretreatment processes, enabling fast determination.

### 2.2. Morphological and Electrochemical Characterization of SNF/ITO

**Figure 2:**
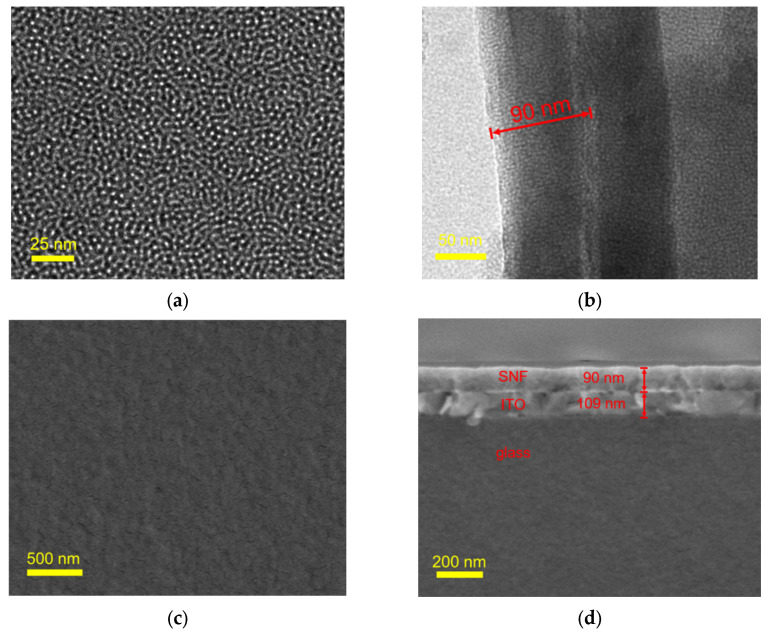
(**a**,**b**) Top-view and cross-sectional TEM images of SNF. (**c**,**d**) SEM image of the surface and the cross-section of SNF/ITO.

The morphology and thickness of SNF were characterized using TEM and SEM. In the top-view TEM image of SNF (Figure 2a), it can be observed that the nanochannel size is uniformly arranged in a worm-like pattern. No defects are observed within the observed range. The pore diameter was measured to be approximately 2.6 nm, with a pore density of ~8 × 10^12^/cm^2^, corresponding to a porosity of approximately 42%. The cross-sectional TEM image (Figure 2b) indicates that the film thickness is approximately 90 nm. Figure 2c,d represent the top-view and the cross-sectional view SEM images of SNF/ITO. It is evident that the SNF surface is smooth with no large particles, demonstrating that no silica nanoparticles are generated during the SNF growth process. The SEM image of cross-section of the SNF/ITO electrode reveals the three-layered structure (Figure 2d). In addition to the glass and ITO layers of the ITO electrode, the SNF layer exhibits a thickness of around 90 nm.

**Figure 3:**
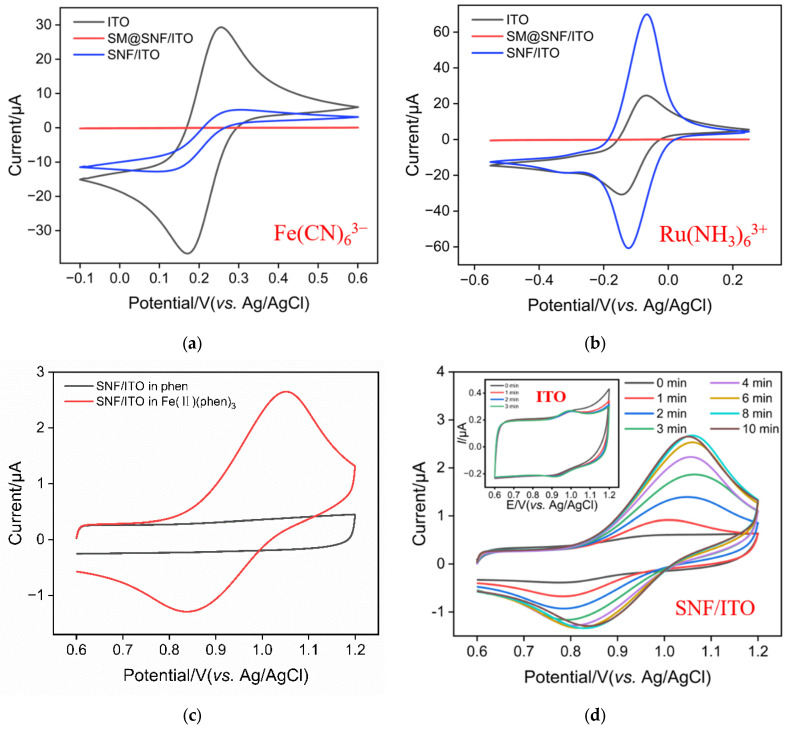
CV curves obtained on ITO, SNF/ITO and SM@SNF/ITO in potassium hydrogen phthalate (KHP) containing 50 μM Fe(CN)_6_^3−^ (**a**) or Ru(NH_3_)_6_^3+^ (**b**). (**c**) CV curves obtained on SNF/ITO in phen (4 μM) or Fe(II)(phen)_3_ (4 μM) solution. (**d**) CV curves obtained on ITO (inset) or the SNF/ITO electrode after stirring in Fe(II)(phen)_3_ (1 μM) for different time.

To confirm the integrity and charge-selective permeability of SNF, redox probes with opposite charges were chosen for cyclic voltammetry (CV) testing. The two redox probes used were the standard anionic probe Fe(CN)_6_^3−^ and the cationic probe Ru(NH_3_)_6_^3+^. As shown in Figure 3a,b, when using SM@SNF/ITO as the working electrode, almost no Faradaic signal is detected in either probe solution because the supporting electrode is covered by an insulating SNF, and SM templates are present inside the nanochannels. Thus, the mass transfer of the redox probes to the underlying electrode is blocked. This indicates that the SNF grown on the ITO electrode surface is complete and has no cracks. After removing the SM template from the nanochannels, the Faradaic signal of the cationic probe Ru(NH_3_)_6_^3+^ on the SNF/ITO electrode significantly increases and is greater than the current signal on the ITO electrode. Consequently, the Faradaic signal of the anionic probe Fe(CN)_6_^4−^ is noticeably suppressed and significantly smaller than that on the ITO electrode. It is evident that SNF exhibits remarkable permeation selectivity for redox probes with different charges. Specifically, the negatively charged nanochannels of SNF electrostatically repel negatively charged small molecules and electrostatically enrich positively charged probes.

### 2.3. Oxidation Potential of Fe(II)(phen)_3_ and Its Enrichment on the SNF/ITO Electrode

To investigate the oxidation–reduction potential of Fe(II)(phen)_3_, CV curves obtained on SNF/ITO electrodes in phenanthroline or Fe(II)(phen)_3_ solution were investigated. As shown in Figure 3c, a significant Faradaic current is observed in the Fe(II)(phen)_3_ solution, while there was almost no Faradaic current in the phenanthroline solution. This confirms that the redox peaks originate from the oxidation of iron atoms in the complex. From the CV curve, it can also be seen that the oxidation reaction of Fe(II)(phen)_3_ occurs at approximately 0.7 V and reaches its peak current at around 1.05 V. 

Figure 3d displayed CV curves obtained on bare ITO or SNF/ITO electrodes in Fe(II)(phen)_3_ solution with stirring for different time. As shown, only a weak signal was observed on the ITO electrode. Even after stirring for 3 min, the signal is closely matched that obtained without stirring, indicating no enrichment in the stirring process. In contrast, the SNF/ITO electrode shows a gradual increase in peak current with increasing stirring time, reaching equilibrium at approximately 8 min of stirring. This current value is approximately 10 times that measured on the ITO electrode, indicating that SNF/ITO exhibits a significant enrichment effect on Fe(II)(phen)_3_. Owing to the abundant silanol groups, SNF carries a negative charge and thus demonstrates a pronounced enrichment effect on the positively charged Fe(II)(phen)_3_, resulting in a significantly increased peak current.

### 2.4. Feasibility Validation for the Electrochemical Sensor and Condition Optimization 

**Figure 4:**
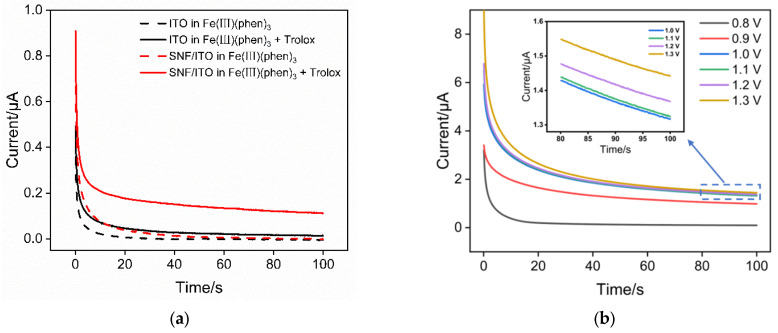
(**a**) *I*–t curves obtained on ITO or SNF/ITO in Fe(III)(phen)_3_ (2 mM) with or without Trolox (1 μM). (**b**) *I*–t curves obtained on SNF/ITO by applying different potentials in Fe(III)(phen)_3_ solution containing 100 μM Trolox.

When the SNF/ITO electrode was immersed in Fe(III)(phen)_3_ solution, Trolox was added and current–time curves (*I*–t) in absence or presence of Trolox were measured using chronoamperometry when a constant oxidative potential (1 V) was applied. Results obtained on the ITO electrode were also measured as control. As shown in Figure 4a, both the bare ITO and SNF/ITO electrodes do not exhibit noticeable signals in absence of Trolox. Obviously, in a solution containing only the oxidized form of Fe(III)(phen)_3_, the application of oxidative potential does not generate Faradaic current. However, upon the addition of Trolox to the solution, a portion of Fe(III)(phen)_3_ is chemically reduced to Fe(II)(phen)_3_. Consequently, the application of a continuous oxidative potential results in a current response, attributed to the electrochemical oxidation of Fe(II)(phen)_3_ in the solution. Thus, when an antioxidant is added, Fe(III)(phen)_3_ is chemically reduced to Fe(II)(phen)_3_, and the oxidation current of Fe(II)(phen)_3_ can be measured by subsequently applying an oxidative potential. This current is positively correlated with the concentration of Fe(II)(phen)_3_ as well as the amount of added antioxidant. In addition, the signal obtained on the SNF/ITO electrode in presence of Trolox increased by 8 fold (0.1115 μA) compared to that of the ITO electrode (0.0137 μA). Thus, the nanochannel layer improve the signal owning to the enrichment of Fe(II)(phen)_3_. Based on this, an electrochemical platform for sensitive TAC determination can be constructed. 

The influence of the applied potential was investigated. As shown in Figure 4b, when a lower potential is applied, the current signal obtained increases with increasing potential. When the potential exceeds +1 V, the current signal stabilizes, indicating that at this potential, all Fe(II)(phen)3 has been oxidized. Therefore, a potential of +1 V was consistently used in subsequent experiments for measuring the oxidation current of Fe(II)(phen)3.

### 2.5. Electrochemical Determination of Trolox

**Figure 5:**
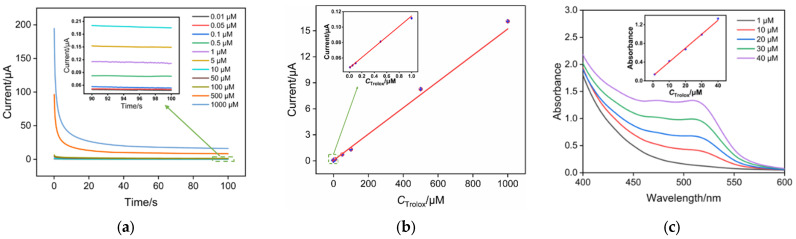
(**a**) *I*–t curves obtained on SNF/ITO electrodes in Fe(III)(phen)_3_ (2 mM) with different concentrations of Trolox. Inset is a magnified view of the *I*–t curve in the low concentration region. (**b**) Calibration curves for Trolox determination. Inset shows a magnified view at low concentrations. (**c**) Spectra obtained for determination of different concentrations of Trolox using the colorimetric method. Inset is the corresponding linear regression curve. Error bars represent the standard deviations of three measurements.

Owing to similar structure to a form of vitamin E (α-tocopherol), Trolox is commonly applied as standard in antioxidant determination. Upon reacting Fe(III)(phen)_3_ with various concentrations of Trolox to form Fe(II)(phen)_3_, a +1 V oxidative potential was applied to measure the electrochemical oxidation current of Fe(II)(phen)_3_ using chronoamperometry. The results are shown in Figure 5a. It is evident that higher concentrations of added Trolox result in higher electrochemical oxidation currents for Fe(II)(phen)_3_. The linear regression curves in Figure 5b demonstrate that there is a good linear relationship between the current (*I*) and Trolox concentration (*C*) in the ranges of 0.01 μM to 1 μM and 1 μM to 1000 μM. At lower Trolox concentrations (0.01 μM to 1 μM), higher sensitivity is achieved (I = 0.0672*C* + 0.0472, *R*^2^ = 0.999). This is due to the more pronounced enrichment effect of SNF/ITO electrodes on Fe(II)(phen)_3_ at lower concentrations. At higher Trolox concentrations, the concentration of Fe(II)(phen)_3_ on the electrode surface tends to saturate, resulting in slightly lower sensitivity compared to that of low concentration (*I* = 0.0152*C* + 0.0535, *R*^2^ = 0.986). The determination limit calculated based on a three signal-to-noise (S/N = 3) is 3.9 nM. The detection of Trolox using the colorimetric method was also investigated. As shown in Figure 5c, the linear determination range was from 1 μM to 40 μM. The linear determination range and the lowest detectable concentration are not as good as that of the electrochemical method. Table S1 (in Supplementary Materials) summarizes the comparison between Trolox detection performance using different methods [12,19,52,53,54]. The LOD obtained using the fabricated sensor is lower than that obtained by differential pulse voltammetry (DPV) detection using a copper-neocuproine complex ([Cu(Nc)_2_]^2+^) probe [52], or a square-wave voltammetry (SWV) detection using a 2,2-diphenyl-1-picrylhydrazyl (DPPH) probe [19], or electrochemiluminescence (ECL) detection based on luminol emitter [53,54], or colorimetric detection using Fe(III)(phen)_3_ probe [12]. Thus, the fabricated sensor also has advantages of convenient fabrication, simple detection, and high sensitivity, demonstrating great potential for TAC determination.

### 2.6. Real Sample Analysis

**Figure 6:**
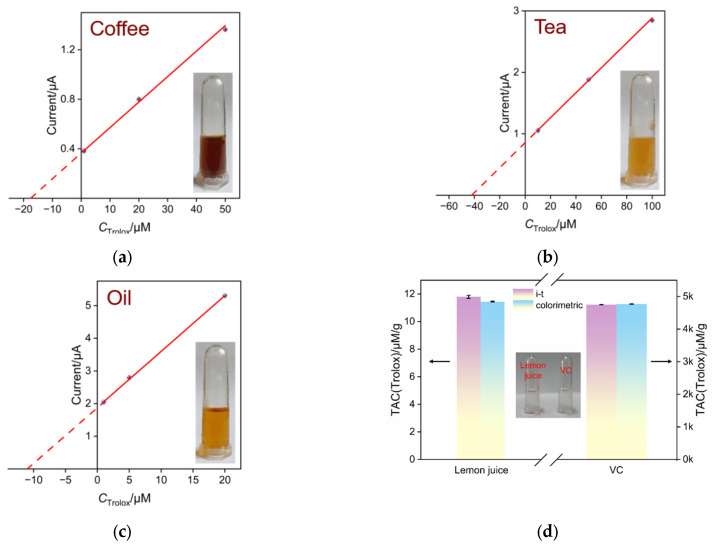
The linear relationship obtained for analysis coffee (**a**), tea (**b**), and sesame oil (**c**) samples. Inset in each figure displays photograph of the analyzed samples. (**d**) TAC of VC tablets or lemon detected by the *I*–t method using the fabricated sensor or standard colorimetric method. The inset shows the digital photo of the detected solution. TAC was calculated according to the weight of the original VC tablet or lemon.

To validate the capability of the prepared electrodes for real sample analysis, coffee, tea, and sesame oil were selected as colored samples to assess their TAC using a standard addition method. TAC was evaluated using Trolox as a reference. Specifically, each sample was directly added to the Fe(III)(phen)_3_ detection solution. Then, different concentrations of Trolox standard solutions were added. The electrochemical signals were measured using SNF/ITO electrodes to obtain a linear regression curve between the electrochemical signal and Trolox concentration. Extrapolating this linear regression curve, the absolute value of its intersection with the *x*-axis (intercept in *x*-axis) corresponds to the equivalent concentration of Trolox of the detected solution. Figure 6a–c showed the linear relationship between the current (I) and the Trolox concentration (C) for different samples. The TAC values in the analysis solution of coffee, tea, and sesame oil samples were found to be 17.62 μM, 43 μM, and 11.18 μM, respectively. These correspond to 251.71 μmol/g, 860 μmol/g, and 22.36 μmol/g with respect to the starting coffee, tea, and sesame oil, respectively. The insets in Figure 6a–c display photos of the analyzed samples, demonstrating that the electrochemical sensor developed in this study can be directly used for the determination of colored samples. In addition, the electrochemical detection obtained on the fabricated sensor was also compared with those obtained from the phen-based colorimetric method. Considering the possible errors in detection of colored samples by the colorimetric method, two colorless samples including the diluted VC tablet solution and diluted lemon juice were detected (inset of Figure 6d). TAC of each sample was measured by the electrochemical method (*I*–t method) using the fabricated sensor or the colorimetric method. As shown in Figure 6d, the results obtained from both detection methods for two samples were nearly identical, demonstrating the reliability of the electrochemical detection of TAC by the sensor constructed in this study.

### 2.7. Regeneration Performance of the Developed Electrochemical Sensor

**Figure 7:**
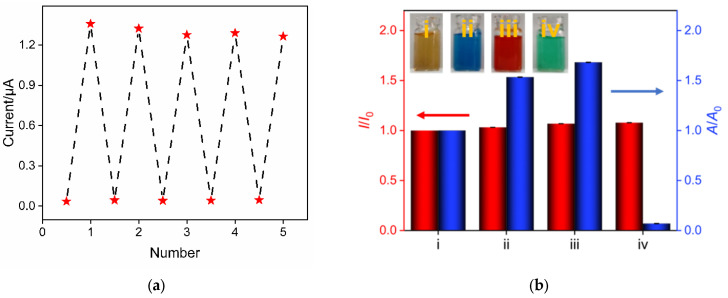
(**a**) The regeneration performance of the developed electrochemical sensor. The signal points below in the figure represent the peak currents measured by the regenerated electrodes in the buffer solution. The signal points above represent the peak currents measured by the regenerated electrodes in the determination solution. (**b**) The relative signal ratio obtained using electrochemical or colorimetric determination. *I* and *I*_0_ and A and A_0_ represent the current signal or absorbance in the presence and absence of colored matrix, respectively. Inset shows photographs of colored samples. The Fe(III)(phen)_3_ detection solution (i) was added with methylene blue (ii), phenol red (iii), or nickel chloride (iv) to simulate colored matrices.

The regeneration performance of the developed sensor was examined. After electrochemical determination in a solution containing Trolox, SNF/ITO electrodes had adsorbed a significant amount of Fe(III)(phen)_3_ or Fe(II)(phen)_3_ molecules in their nanochannels. Subsequently, they were immersed in an HCl–ethanol solution and stirred for 3 min to remove the adsorbed molecules. After washing the electrodes, electrochemical determination was carried out in a buffer solution or the detection solution. As shown in Figure 7a, the regenerated electrode exhibits minimal current signal in the buffer solution, demonstrating that the adsorbed substances on the electrode could be thoroughly removed through a simple washing process. When the regenerated electrode is re-used in the detection solution, the signal remains nearly unchanged. After five regenerations, the relative standard deviation (RSD) of the measured current signal is 2.9%, indicating good regeneration performance.

### 2.8. Comparison between Electrochemical and Colorimetric Methods for Analyze Colored Samples

The determination performance obtained using the constructed electrochemical sensor or common phen-based colorimetric determination was compared for Trolox determination in colored samples. The results are shown in Figure 7b. The Fe(III)(phen)_3_ detection solution (i) was added with methylene blue (ii), phenol red (iii), or nickel chloride (iv) to simulate colored matrices, and the determination performance of Trolox in these colored matrices was investigated. *I*_0_ and *I* represent the current signals obtained in the absence and presence of colored substances, while A_0_ and A represent the absorbance obtained in the absence and presence of colored matrices. It can be observed that the presence of colored substances significantly affects the determination results of the colorimetric method, while the results of electrochemical determination are hardly affected. This demonstrates that our determination method can be used for accurate determination of colored samples.

## 3. Materials and Methods

### 3.1. Chemicals and Materials

Trolox, phenoline hydrochloride (phen), ammonium ferric sulfate dodecahydrate (NH4Fe(SO_4_)_2_•12H_2_O), ferrous chloride (FeCl_2_), tetraethoxysilane (TEOS, 98%), potassium ferricyanide (K_3_[Fe(CN)_6_]), ruthenium hexamethonium chloride (Ru(NH_3_)_6_Cl_3_), and 3-aminopropyltriethoxysilane (GPTMS) were purchased from Aladdin Biochemical Technology Co., Ltd. (Shanghai, China). Cetyltrimethylammonium bromide (CTAB), acetone, ethanol (99.8%) and concentrated hydrochloric acid (HCl, 38%) were purchased from Kermel Chemical Reagent Company (Tianjin, China). All reagents were used without further purification. To synthesize Fe(III)(phen)_3_, a solution of NH_4_Fe(SO_4_)_2_ (3 mL, 0.5 M, dissolved in 1 M HCl) was combined with phen solution (15 mL, 0.4 M). To ensure complete coordination of the iron ions, an excess of the phen solution was employed. The obtained solution was diluted to 2 mM (based on the molar quantity of the iron ions) before use. The Trolox stock solution (0.1 M) was prepared in ethanol and diluted to achieve desired concentrations.

Indium tin oxide (ITO) conductive glass (square resistance <17 Ω/sq, ITO thickness: 100 ± 20 nm) was purchased from Kaiwei Optoelectronics Technology Co., Ltd. (Zhuhai, China). A simple pretreatment was carried out before use. Briefly, ITO glass was cut to a size of 2.5 cm × 5 cm, sonicated for 60 min in NaOH (1 M), followed by ultrasonic cleaning with acetone, ethanol and deionized water for 30 min in turn.

### 3.2. Characteriaztions and Instrumentations

Transmission electron microscopy (TEM) and scanning electron microscopy (SEM) were employed to examine the structural characteristics of the synthesized SNF material. TEM analysis was conducted using a Hitachi HT7700 microscope operating at an acceleration voltage of 200 kV. For TEM sample preparation, the SNF layer was meticulously scraped off the surface of an ITO substrate using a sharp blade and subsequently dispersed in ethanol via ultrasonication. The resulting dispersion was carefully deposited onto a copper grid, allowed to dry under a lamp, and then subjected to TEM imaging. SEM characterization was carried out on a Zeiss ULTRA 55 microscope using an acceleration voltage of 5 kV. UV–vis absorption spectroscopy (UV–vis) was performed with a Shimadzu UV-2450 spectrometer. Electrochemical characterization, including cyclic voltammetry (CV) or amperometry (*I*–t) test, was conducted using a CHI660D electrochemical workstation (Chinstrument, Shanghai, China). These electrochemical measurements were carried out with three commercial electrodes, featuring an Ag/AgCl reference electrode saturated with potassium chloride as the reference electrode, a platinum wire as the counter electrode, and ITO- or SNF-modified electrodes as the working electrode.

### 3.3. Preparation of the SNF-Modified ITO Electrode

SNF modification layer were grown through the Stöber solution growth method on the supporting ITO electrode [51,55,56]. Specifically, CTAB (0.160 g) was completely dissolved in a mixture of 70 mL of ethanol and 30 mL of water. Subsequently, 100 μL of 10% ammonia and 80 μL of TEOS were added to the solution under continuous stirring to obtain a clear and transparent precursor solution without any visible bubbles. Then, clean ITO glass (2.5 cm × 5 cm) was immersed in the precursor solution. SNF was grown at 60 °C for 24 h. After the growth process was completed, the electrode was removed, thoroughly rinsed with deionized water, dried using nitrogen gas. Then, the obtained electrode was placed in an oven at 100 °C overnight for aging, resulting in a SNF-modified electrode containing micelle (SM) template within the nanochannels. The obtained electrode was denoted as SM@SNF/ITO. The electrode was then cut, and its area was controlled to 0.5 cm × 1 cm using insulating glue. To remove the SM template, the SM@SNF/ITO electrode was immersed in a 0.1 M HCl–ethanol solution and stirred for 5 min. The resulting electrode featuring open channels was designated as SNF/ITO.

### 3.4. Electrochemical Determination of Trolox

Electrochemical determination was performed using a Fe(III)(phen)_3_ solution (2 mM) as the determination solution. Specifically, different concentrations of Trolox were introduced into the Fe(III)(phen)_3_ solution. Then, a fixed potential of +1 V was applied, and the oxidation current produced by the chemically reduced Fe(II)(phen)_3_ was measured using chronoamperometry.

### 3.5. Real Sample Analysis

In the analysis of real samples, coffee (Shefei Brand, obtained from local market, Hangzhou, China), tea leaves (Anji Brand, obtained from local market, Hangzhou, China) and sesame oil (Golden Brand, Nanjing, China) require no complex pretreatment. Specifically, coffee was prepared by dissolving coffee powder in deionized water (1 g/100 mL). Tea leaves were soaked in distilled water (10 g/100 mL) for 30 min, and the supernatant was collected. Sesame oil was dispersed in ethanol using ultrasonication (10 g/100 mL). These samples were mixed directly with the Fe(III)(phen)_3_ determination solution for analysis. TAC in the VC tablet or lemon was also analyzed. Specifically, the VC tablet was dissolved in ultrapure water and diluted to 1.1 mg/mL. The lemon juice squeezed out from lemon was diluted with ultrapure water to 0.1 g/mL. TAC was then detected by the standard addition method using the electrochemical method constructed in this work or the phen-based colorimetric method using characteristic absorption of Fe(II)(phen)_3_ at 520 nm. TAC was calculated based on the weight of the initial sample and expressed as the Trolox equivalent per gram of sample (μM/g). 

## 4. Conclusions

In summary, a rapid and sensitive electrochemical platform was established for determination of TAC in food samples based on nanochannel-modified electrodes. Employing the Stöber solution growth method, multiple nanochannel-modified electrodes (SNF/ITO) can be readily prepared on low-cost and easily obtainable ITO electrodes. The used Fe(III)(phen)_3_ probe can be chemically reduced to Fe(II)(phen)_3_ in the presence of antioxidants, which can be electrochemically oxidized at a high potential. Determination of antioxidants was achieved through the measurement of the oxidation current of Fe(II)(phen)_3_. Due to the electrostatic enrichment of Fe(III)(phen)_3_ by the negatively charged nanochannels of SNF, the SNF/ITO electrode exhibits significantly enhanced electrochemical signals as well as the detection sensitivity. Additionally, the size-exclusion effect of the ultrasmall nanochannels of SNF and the insulating nature of the SiO_2_ structure provide the SNF/ITO electrode with good anti-interference capability, enabling direct detection of colored samples. Thus, the constructed electrochemical sensor exhibits good determination performance compared to traditional colorimetric methods. The fabricated sensor can be directly applied to the measurement of TAC in colored samples such as coffee, tea, and edible oils. Owing to the easy fabrication, fast detection, and high sensitivity, the developed electrochemical sensor holds great potential for TAC determination in food samples without the need for complex sample pretreatment.

## Data Availability

The data presented in this study are available on request from the corresponding author.

## Supplementary Materials

The following supporting information can be downloaded at: https://www.mdpi.com/article/10.3390/molecules29112423/s1, Table S1: Comparison between Trolox detection performance using different methods.
