# Supplementary material for: Direct and Sensitive Electrochemical Determination of Total Antioxidant Capacity in Foods Using Nanochannel-Based Enrichment of Redox Probes
Source: Molecules. 2024 May 21;29(11):2423. doi: 10.3390/molecules29112423 (PMC11173714; doi:10.3390/molecules29112423)
Supplement: Supplementary file 1 [file molecules-29-02423-s001.zip › molecules-2967427-supplementary.pdf]

molecules

MDPI

Supplementary Materials

Table S1. Comparison between Trolox detection performance using different methods.

Electrode	Probe	Method	Linear range (μM)	LOD (μM)	Real samples	Ref.
SPMEs	$[Cu(Nc)_2]^{2+}$	DPV	0.4–1.2	0.2	Tea	[52]
G/PEDOT:PSS/SPCE	DPPH	SWV	5–30	0.59	Thai herb/Herbal beverage	[19]
SPCE	luminol	ECL	0–2000	-	Mixed vegetable and fruit juice/Tea/Coffee	[53]
HEO/N-G-900/GCE	luminol	ECL	0.001–25	0.001	Tea/Vitamin C	[54]
-	$Fe(III)(phen)_3$	colorimetric	6.25–31.25	0.26	Fruit juices	[12]
SNF/ITO	$Fe(III)(phen)_3$	i-t	0.01–1000	0.0039	Tea/Coffee/Oil/ Lemon juice/Vitamin C	This work

SPMEs: screen-printed microelectrodes; $[Cu(Nc)_2]^{2+}$: copper-neocuproine complex; DPV: differential pulse voltammetry; G: graphene; PEDOT: poly(3,4-ethylenedioxythiophene); PSS: poly(styrenesulfonate); SPCE: screen-printed carbon electrode; DPPH: 2,2-diphenyl-1-picrylhydrazyl; SWV: square wave voltammetry; ECL: electrochemiluminescence; HEO: high-entropy oxide; N-G: nitrogen-doped graphene; GCE: glassy carbon electrode.
